# 1,3-Bis[2-(2-oxo-1,3-oxazolidin-3-yl)eth­yl]-1*H*-benzimidazol-2(3*H*)-one

**DOI:** 10.1107/S1600536810052141

**Published:** 2011-01-12

**Authors:** Younes Ouzidan, Youssef Kandri Rodi, Frank R. Fronczek, Ramaiyer Venkatraman, Lahcen El Ammari, El Mokhtar Essassi

**Affiliations:** a Laboratoire de Chimie Organique Appliquée, Université Sidi Mohamed Ben Abdallah, Faculté des Sciences et Techniques, Route d’Immouzzer, BP 2202 Fès, Morocco; b Department of Chemistry, Louisiana State University, Baton Rouge, LA 70803, USA; c Department of Chemistry and Biochemistry, Jackson State University, Jackson, MS 39217, USA; d Laboratoire de Chimie du Solide Appliquée, Faculté des Sciences, Université Mohammed V-Agdal, Avenue Ibn Battouta, BP 1014, Rabat, Morocco; e INANOTECH (Institute of Nanomaterials and Nanotechnology), MASCiR, Avenue de l’Armée Royale, Rabat, Morocco

## Abstract

The mol­ecular structure of the title compound, C_17_H_20_N_4_O_5_, contains a central fused-ring system, comprised of six- and five-membered rings. This unit is linked by C_2_ chains to two 2-oxo-1,3-oxazolidine five-membered rings. The central fused-ring system is essentially planar, with a maximum deviation of 0.008 (1) Å from the mean plane. Both oxazolidine five-membered rings are also nearly planar, with maximum deviations of 0.090 (1) and 0.141 (1) Å.

## Related literature

For the pharmacological and biochemical properties of oxazolidin-2-ones, see: Gribkoff *et al.* (1994 ▶); Olesen *et al.* (1994 ▶); Soderlind *et al.* (1999 ▶). For their anti­bacterial activity, see: Diekema & Jones (2000 ▶); Mukhtar & Wright (2005 ▶). For related structures, see: Ouzidan *et al.* (2010 ▶); Matsunaga *et al.* (2005 ▶); Evans *et al.* (1993 ▶); Caleb *et al.* (2009 ▶); Ahoya *et al.* (2010 ▶); Bel-Ghacham *et al.* (2010 ▶); Alsubari *et al.* (2009 ▶).
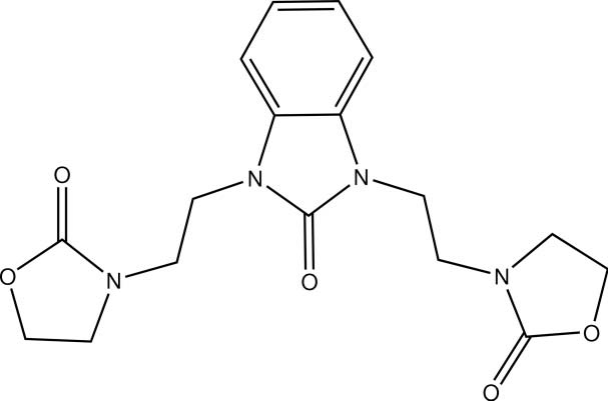


## Experimental

### 

#### Crystal data

C_17_H_20_N_4_O_5_
*M* *_r_* = 360.37
Monoclinic, 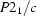

*a* = 10.5331 (10) Å
*b* = 10.9647 (10) Å
*c* = 14.5541 (14) Å
β = 103.258 (5)°
*V* = 1636.1 (3) Å^3^
*Z* = 4
Cu *K*α radiation
μ = 0.92 mm^−1^
*T* = 90 K
0.30 × 0.28 × 0.18 mm

#### Data collection

Bruker APEXII CCD diffractometer
Absorption correction: multi-scan (*SADABS*; Bruker, 2005 ▶) *T*_min_ = 0.770, *T*_max_ = 0.852
15549 measured reflections
2914 independent reflections
2842 reflections with *I* > 2σ(*I*)
*R*_int_ = 0.024

#### Refinement

*R*[*F* ^2^ > 2σ(*F* ^2^)] = 0.030
*wR*(*F* ^2^) = 0.074
*S* = 1.05
2914 reflections
235 parameters
H-atom parameters constrained
Δρ_max_ = 0.23 e Å^−3^
Δρ_min_ = −0.19 e Å^−3^

### 

Data collection: *APEX2* (Bruker, 2005 ▶); cell refinement: *SAINT* (Bruker, 2005 ▶); data reduction: *SAINT*; program(s) used to solve structure: *SHELXS97* (Sheldrick, 2008 ▶); program(s) used to refine structure: *SHELXL97* (Sheldrick, 2008 ▶); molecular graphics: *ORTEP-3 for Windows* (Farrugia, 1997 ▶); software used to prepare material for publication: *WinGX* (Farrugia, 1999 ▶).

## Supplementary Material

Crystal structure: contains datablocks I, global. DOI: 10.1107/S1600536810052141/fj2373sup1.cif

Structure factors: contains datablocks I. DOI: 10.1107/S1600536810052141/fj2373Isup2.hkl

Additional supplementary materials:  crystallographic information; 3D view; checkCIF report

## Figures and Tables

**Fig. 1.**
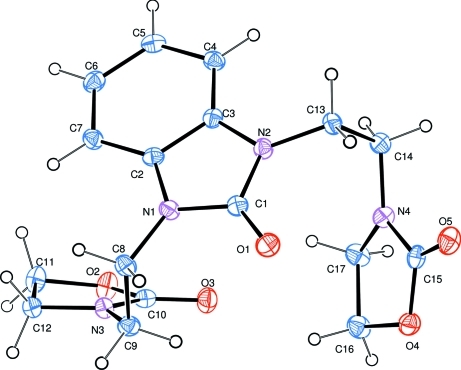
Molecular structure of the title compound with the atom-labelling scheme. Displacement ellipsoids are drawn at the 50% probability level. H atoms are represented as small circles.

## supplementary crystallographic information

### Comment

Benzimidazol-2-one derivatives are useful heterocyclic building blocks and are prominent structural elements of compounds presenting a wide variety of pharmacological and biochemical properties(Gribkoff *et al.* (1994); Olesen *et al.* (1994); Soderlind *et al.* (1999).

Also oxazolidin-2-ones are a very important class of heterocyclic compounds and their derivatives have attracted attention in various areas of drug development for antibacterial activity (Diekema & Jones, 2000); Mukhtar & Wright (2005). Some oxazolidin-2-ones have been used as chiral auxiliaries in a wide range of asymmetric reactions (Evans *et al.*, 1993); Matsunaga *et al.* (2005).

In the previous works, we have studied the crystal structure of several heterocyclic systems containng oxazolidin-2-one (Ouzidan *et al.* (2010); Caleb *et al.* (2009); Ahoya *et al.* (2010); Bel-Ghacham *et al.* (2010); Alsubari *et al.* (2009)). In this work, we have synthesized benzimidazol-2-one possessing Oxazolidin-2-one ring by action of bis(2-chloroethyl)amine hydrochloride with 1*H*-benzo[*d*]imidazol-2(3*H*)-one using same conditions, the reaction provided the title compound (Scheme 1).

The 1,3-bis(2-(2-oxo-oxazolidin-3-yl)ethyl)-1*H*-benzimidazol -\2(3*H*)-one molecule structure is built up from two fused six-and five-membered rings linked to two chains of five-membered rings by ethylene groups as schown in Fg.1. The fused-ring system is essentially planar, with a maximum deviation of 0.008 (1) Å and -0.008 (1) Å for C1 and C7 or N2 respectyvely. The dihedral angle between them does not exceed 0.23 (6)°. The both five-membered rings (2-oxo-oxazolidine) are almost planar with maximum deviation of -0.090 (1) Å and -0.141 (1) Å for C11 and C16 respectyvely. Their puckering parameters are Q2 = 0.1498 (2) Å and φ2 = 131.6 (5)° for (O2C10N3C12C11) and Q2 = 0.2343 (2) Å and φ2 = -49.4 (3)° for (O4C15N4C17C16). The dihedral angles between each of them and the fused rings are 43.02 (5)° (O2C10N3C12C11) and 49.12 (6)° (O4C15N4C17C16). The torsion angles C1 N1 C8 C9 and C1 N2 C13 C14 are -87.85 (2)° and 101.00 (2)° respectively. These values show the strong asymmetry of the molecule.

### Experimental

To 1*H*-benzo[*d*]imidazol-2(3*H*)-one (0,2 g, 1,5 mmol), potassium carbonate (0,82 g, 6 mmol), and tetra-n-butylammonium bromide (0.1 g, 0,3 mmol) in DMF (15 ml) was added bis(2-chloroethyl)amine hydrochloride (0,64 g, 3,58 mmol). The mixture was heated for 48 h. After the completion of the reaction (as monitored by TLC), the inorganic material salt was filtered and the solvent was removed under reduced pressure. The residue was purified by column chromatography on silica gel by using (ethanol/ethyl acetate: 1/4) as eluent. Colorless crystals were isolated when the solvent was allowed to evaporate.

### Refinement

H atoms were located in a difference map and treated as riding with C—H = 0.93 Å for all H atoms with *U*_iso_(H) = 1.2 *U*_eq_(aromatic, methine).

### Crystal data

**Table:**

C_17_H_20_N_4_O_5_	*F*(000) = 760
*M**_r_* = 360.37	*D*_x_ = 1.463 Mg m^−^^3^
Monoclinic, *P*2_1_/*c*	Cu *K*α radiation, λ = 1.54178 Å
Hall symbol: -P 2ybc	Cell parameters from 9916 reflections
*a* = 10.5331 (10) Å	θ = 4.0–68.1°
*b* = 10.9647 (10) Å	µ = 0.92 mm^−^^1^
*c* = 14.5541 (14) Å	*T* = 90 K
β = 103.258 (5)°	Fragment, colourless
*V* = 1636.1 (3) Å^3^	0.30 × 0.28 × 0.18 mm
*Z* = 4	

### Data collection

**Table:**

Bruker APEXII CCD diffractometer	2914 independent reflections
Radiation source: fine-focus sealed tube	2842 reflections with *I* > 2σ(*I*)
graphite	*R*_int_ = 0.024
φ and ω scans	θ_max_ = 68.2°, θ_min_ = 4.3°
Absorption correction: multi-scan (*SADABS*; Bruker, 2005)	*h* = −12→9
*T*_min_ = 0.770, *T*_max_ = 0.852	*k* = −12→13
15549 measured reflections	*l* = −17→17

### Refinement

**Table:**

Refinement on *F*^2^	Primary atom site location: structure-invariant direct methods
Least-squares matrix: full	Secondary atom site location: difference Fourier map
*R*[*F*^2^ > 2σ(*F*^2^)] = 0.030	Hydrogen site location: inferred from neighbouring sites
*wR*(*F*^2^) = 0.074	H-atom parameters constrained
*S* = 1.05	*w* = 1/[σ^2^(*F*_o_^2^) + (0.0315*P*)^2^ + 0.8105*P*] where *P* = (*F*_o_^2^ + 2*F*_c_^2^)/3
2914 reflections	(Δ/σ)_max_ < 0.001
235 parameters	Δρ_max_ = 0.23 e Å^−^^3^
0 restraints	Δρ_min_ = −0.19 e Å^−^^3^

### Special details

**Table:**

Geometry. All e.s.d.'s (except the e.s.d. in the dihedral angle between two l.s. planes) are estimated using the full covariance matrix. The cell e.s.d.'s are taken into account individually in the estimation of e.s.d.'s in distances, angles and torsion angles; correlations between e.s.d.'s in cell parameters are only used when they are defined by crystal symmetry. An approximate (isotropic) treatment of cell e.s.d.'s is used for estimating e.s.d.'s involving l.s. planes.
Refinement. Refinement of *F*^2^ against ALL reflections. The weighted *R*-factor *wR* and goodness of fit *S* are based on *F*^2^, conventional *R*-factors *R* are based on *F*, with *F* set to zero for negative *F*^2^. The threshold expression of *F*^2^ > σ(*F*^2^) is used only for calculating *R*-factors(gt) *etc*. and is not relevant to the choice of reflections for refinement. *R*-factors based on *F*^2^ are statistically about twice as large as those based on *F*, and *R*- factors based on ALL data will be even larger.

### Fractional atomic coordinates and isotropic or equivalent isotropic displacement parameters (Å^2^)

**Table:**

	*x*	*y*	*z*	*U*_iso_*/*U*_eq_	
O1	0.51156 (8)	0.90454 (8)	0.65034 (6)	0.01864 (19)	
O2	0.81349 (8)	0.63845 (8)	0.39970 (6)	0.0221 (2)	
O3	0.61653 (8)	0.68768 (8)	0.42496 (6)	0.0209 (2)	
O4	0.22108 (8)	0.77982 (8)	0.47938 (6)	0.01792 (19)	
O5	0.18927 (8)	0.77876 (8)	0.62760 (6)	0.0217 (2)	
N1	0.71846 (9)	0.83091 (9)	0.64775 (7)	0.0145 (2)	
N2	0.58627 (9)	0.71116 (9)	0.70549 (7)	0.0143 (2)	
N3	0.79091 (9)	0.81926 (9)	0.46197 (7)	0.0149 (2)	
N4	0.33456 (9)	0.64839 (9)	0.58183 (7)	0.0158 (2)	
C1	0.59511 (11)	0.82469 (11)	0.66601 (8)	0.0143 (2)	
C2	0.78498 (11)	0.72200 (10)	0.67384 (8)	0.0142 (2)	
C3	0.70112 (11)	0.64616 (11)	0.71027 (7)	0.0137 (2)	
C4	0.73829 (11)	0.52998 (11)	0.74338 (8)	0.0157 (2)	
H4	0.6828	0.4800	0.7679	0.019*	
C5	0.86290 (11)	0.49150 (11)	0.73819 (8)	0.0174 (3)	
H5	0.8907	0.4137	0.7591	0.021*	
C6	0.94645 (11)	0.56687 (11)	0.70237 (8)	0.0176 (3)	
H6	1.0288	0.5384	0.7000	0.021*	
C7	0.90915 (11)	0.68422 (11)	0.66999 (8)	0.0162 (2)	
H7	0.9653	0.7350	0.6468	0.019*	
C8	0.76194 (11)	0.93510 (10)	0.60123 (8)	0.0159 (2)	
H8A	0.8558	0.9432	0.6227	0.019*	
H8B	0.7223	1.0086	0.6190	0.019*	
C9	0.72673 (11)	0.92265 (11)	0.49385 (8)	0.0162 (2)	
H9A	0.6330	0.9132	0.4724	0.019*	
H9B	0.7516	0.9966	0.4659	0.019*	
C10	0.72954 (11)	0.71504 (11)	0.42958 (8)	0.0160 (2)	
C11	0.94356 (12)	0.69048 (12)	0.42409 (9)	0.0210 (3)	
H11A	0.9846	0.6861	0.3709	0.025*	
H11B	0.9977	0.6475	0.4771	0.025*	
C12	0.92429 (11)	0.82316 (11)	0.45003 (8)	0.0170 (3)	
H12A	0.9852	0.8466	0.5080	0.020*	
H12B	0.9329	0.8785	0.3998	0.020*	
C13	0.47432 (11)	0.67173 (11)	0.74094 (8)	0.0156 (2)	
H13A	0.4262	0.7430	0.7533	0.019*	
H13B	0.5050	0.6290	0.8002	0.019*	
C14	0.38313 (11)	0.58849 (11)	0.67225 (8)	0.0169 (2)	
H14A	0.4293	0.5147	0.6628	0.020*	
H14B	0.3102	0.5655	0.6989	0.020*	
C15	0.24555 (11)	0.73773 (11)	0.57032 (8)	0.0159 (2)	
C16	0.31809 (12)	0.72690 (11)	0.43458 (9)	0.0198 (3)	
H16A	0.3889	0.7837	0.4355	0.024*	
H16B	0.2794	0.7048	0.3697	0.024*	
C17	0.36658 (12)	0.61423 (11)	0.49327 (8)	0.0197 (3)	
H17A	0.3204	0.5413	0.4665	0.024*	
H17B	0.4596	0.6023	0.5006	0.024*	

### Atomic displacement parameters (Å^2^)

**Table:**

	*U*^11^	*U*^22^	*U*^33^	*U*^12^	*U*^13^	*U*^23^
O1	0.0189 (4)	0.0159 (4)	0.0217 (4)	0.0033 (3)	0.0056 (3)	0.0003 (3)
O2	0.0169 (4)	0.0205 (5)	0.0288 (5)	−0.0003 (3)	0.0049 (3)	−0.0081 (4)
O3	0.0160 (4)	0.0201 (5)	0.0261 (5)	−0.0024 (3)	0.0038 (3)	−0.0008 (4)
O4	0.0165 (4)	0.0186 (4)	0.0190 (4)	0.0030 (3)	0.0048 (3)	0.0007 (3)
O5	0.0178 (4)	0.0263 (5)	0.0222 (4)	0.0031 (4)	0.0069 (3)	−0.0052 (4)
N1	0.0156 (5)	0.0134 (5)	0.0151 (5)	0.0001 (4)	0.0047 (4)	0.0010 (4)
N2	0.0135 (5)	0.0143 (5)	0.0155 (5)	0.0002 (4)	0.0044 (4)	0.0001 (4)
N3	0.0145 (5)	0.0147 (5)	0.0159 (5)	−0.0007 (4)	0.0042 (4)	−0.0003 (4)
N4	0.0161 (5)	0.0158 (5)	0.0155 (5)	0.0018 (4)	0.0037 (4)	−0.0017 (4)
C1	0.0158 (6)	0.0145 (6)	0.0123 (5)	−0.0005 (5)	0.0025 (4)	−0.0022 (4)
C2	0.0164 (6)	0.0143 (6)	0.0108 (5)	−0.0001 (5)	0.0010 (4)	−0.0011 (4)
C3	0.0139 (5)	0.0159 (6)	0.0111 (5)	−0.0006 (4)	0.0020 (4)	−0.0023 (4)
C4	0.0184 (6)	0.0160 (6)	0.0125 (5)	−0.0018 (5)	0.0031 (4)	0.0001 (4)
C5	0.0211 (6)	0.0153 (6)	0.0148 (5)	0.0034 (5)	0.0019 (4)	0.0014 (4)
C6	0.0157 (6)	0.0212 (6)	0.0158 (5)	0.0038 (5)	0.0033 (4)	−0.0002 (5)
C7	0.0163 (6)	0.0188 (6)	0.0141 (5)	−0.0013 (5)	0.0047 (4)	0.0001 (4)
C8	0.0181 (6)	0.0118 (6)	0.0183 (6)	−0.0016 (5)	0.0054 (4)	−0.0003 (4)
C9	0.0179 (6)	0.0133 (6)	0.0175 (6)	0.0020 (5)	0.0042 (4)	0.0017 (4)
C10	0.0177 (6)	0.0163 (6)	0.0136 (5)	0.0017 (5)	0.0024 (4)	0.0017 (4)
C11	0.0149 (6)	0.0240 (7)	0.0241 (6)	0.0000 (5)	0.0049 (5)	−0.0046 (5)
C12	0.0146 (6)	0.0201 (6)	0.0169 (6)	−0.0007 (5)	0.0046 (4)	0.0016 (5)
C13	0.0147 (6)	0.0172 (6)	0.0159 (5)	−0.0010 (5)	0.0057 (4)	−0.0003 (5)
C14	0.0166 (6)	0.0161 (6)	0.0179 (6)	−0.0008 (5)	0.0038 (4)	0.0012 (5)
C15	0.0121 (5)	0.0165 (6)	0.0183 (6)	−0.0028 (5)	0.0020 (4)	−0.0030 (5)
C16	0.0215 (6)	0.0197 (6)	0.0206 (6)	0.0029 (5)	0.0097 (5)	0.0006 (5)
C17	0.0246 (6)	0.0175 (6)	0.0197 (6)	0.0033 (5)	0.0106 (5)	−0.0001 (5)

### Geometric parameters (Å, °)

**Table:**

O1—C1	1.2252 (14)	C5—H5	0.9300
O2—C10	1.3612 (14)	C6—C7	1.3955 (17)
O2—C11	1.4510 (15)	C6—H6	0.9300
O3—C10	1.2144 (14)	C7—H7	0.9300
O4—C15	1.3692 (14)	C8—C9	1.5273 (16)
O4—C16	1.4531 (14)	C8—H8A	0.9700
O5—C15	1.2147 (14)	C8—H8B	0.9700
N1—C1	1.3862 (15)	C9—H9A	0.9700
N1—C2	1.3926 (15)	C9—H9B	0.9700
N1—C8	1.4545 (15)	C11—C12	1.5282 (17)
N2—C1	1.3831 (15)	C11—H11A	0.9700
N2—C3	1.3921 (15)	C11—H11B	0.9700
N2—C13	1.4570 (14)	C12—H12A	0.9700
N3—C10	1.3439 (16)	C12—H12B	0.9700
N3—C9	1.4496 (15)	C13—C14	1.5215 (16)
N3—C12	1.4552 (14)	C13—H13A	0.9700
N4—C15	1.3396 (15)	C13—H13B	0.9700
N4—C17	1.4542 (15)	C14—H14A	0.9700
N4—C14	1.4546 (15)	C14—H14B	0.9700
C2—C7	1.3853 (16)	C16—C17	1.5220 (17)
C2—C3	1.4027 (16)	C16—H16A	0.9700
C3—C4	1.3862 (17)	C16—H16B	0.9700
C4—C5	1.3971 (17)	C17—H17A	0.9700
C4—H4	0.9300	C17—H17B	0.9700
C5—C6	1.3927 (17)		
			
C10—O2—C11	108.99 (9)	C8—C9—H9B	109.2
C15—O4—C16	107.65 (9)	H9A—C9—H9B	107.9
C1—N1—C2	109.92 (9)	O3—C10—N3	128.03 (11)
C1—N1—C8	122.48 (10)	O3—C10—O2	122.02 (11)
C2—N1—C8	127.44 (10)	N3—C10—O2	109.95 (10)
C1—N2—C3	109.94 (9)	O2—C11—C12	105.27 (9)
C1—N2—C13	123.46 (9)	O2—C11—H11A	110.7
C3—N2—C13	126.51 (10)	C12—C11—H11A	110.7
C10—N3—C9	123.71 (10)	O2—C11—H11B	110.7
C10—N3—C12	112.52 (10)	C12—C11—H11B	110.7
C9—N3—C12	123.31 (10)	H11A—C11—H11B	108.8
C15—N4—C17	112.04 (10)	N3—C12—C11	100.84 (9)
C15—N4—C14	122.29 (10)	N3—C12—H12A	111.6
C17—N4—C14	125.49 (10)	C11—C12—H12A	111.6
O1—C1—N2	127.34 (11)	N3—C12—H12B	111.6
O1—C1—N1	126.44 (11)	C11—C12—H12B	111.6
N2—C1—N1	106.22 (9)	H12A—C12—H12B	109.4
C7—C2—N1	131.76 (11)	N2—C13—C14	112.67 (9)
C7—C2—C3	121.35 (11)	N2—C13—H13A	109.1
N1—C2—C3	106.88 (10)	C14—C13—H13A	109.1
C4—C3—N2	131.36 (11)	N2—C13—H13B	109.1
C4—C3—C2	121.60 (11)	C14—C13—H13B	109.1
N2—C3—C2	107.03 (10)	H13A—C13—H13B	107.8
C3—C4—C5	116.91 (11)	N4—C14—C13	111.20 (10)
C3—C4—H4	121.5	N4—C14—H14A	109.4
C5—C4—H4	121.5	C13—C14—H14A	109.4
C6—C5—C4	121.54 (11)	N4—C14—H14B	109.4
C6—C5—H5	119.2	C13—C14—H14B	109.4
C4—C5—H5	119.2	H14A—C14—H14B	108.0
C5—C6—C7	121.36 (11)	O5—C15—N4	128.51 (11)
C5—C6—H6	119.3	O5—C15—O4	121.68 (11)
C7—C6—H6	119.3	N4—C15—O4	109.79 (10)
C2—C7—C6	117.21 (11)	O4—C16—C17	104.61 (9)
C2—C7—H7	121.4	O4—C16—H16A	110.8
C6—C7—H7	121.4	C17—C16—H16A	110.8
N1—C8—C9	112.19 (9)	O4—C16—H16B	110.8
N1—C8—H8A	109.2	C17—C16—H16B	110.8
C9—C8—H8A	109.2	H16A—C16—H16B	108.9
N1—C8—H8B	109.2	N4—C17—C16	99.92 (9)
C9—C8—H8B	109.2	N4—C17—H17A	111.8
H8A—C8—H8B	107.9	C16—C17—H17A	111.8
N3—C9—C8	112.07 (9)	N4—C17—H17B	111.8
N3—C9—H9A	109.2	C16—C17—H17B	111.8
C8—C9—H9A	109.2	H17A—C17—H17B	109.5
N3—C9—H9B	109.2		
